# Macro and micro-elements concentrations in Calligonum comosum wild grazing plant through its growth period

**DOI:** 10.1016/j.sjbs.2021.07.084

**Published:** 2021-08-02

**Authors:** Mohamed I. Alzarah

**Affiliations:** Department of Environmental and Natural Resources, College of Agricultural and Food Sciences, King Faisal University, P.O. Box 420, Al-Hassa 31982, Saudi Arabia

**Keywords:** *Calligonum* comosum, Grazing plants, Grazing animal, Macro elements, Micro elements, Leaves, Stem, Root, Al-Ahsa Governorate, Kingdom of Saudi Arabia

## Abstract

In this study, the change in the content of the macro and micro elements in the growing wild grazing plant of *Calligonum comosum* was tracked at the Research and Training Station of King Faisal University in Al-Hassa Governorate, Kingdom of Saudi Arabia. Mineral elements were estimated in aerial parts (plant as a whole, leaves and stem) from January-April 2020. The results showed that the concentration of nitrogen, phosphorus and potassium in the plant as a whole plant > leaves > roots, while the concentrations of calcium, magnesium, manganese, zinc and copper elements in the leaves was higher than other parts whereas the concentrations of these elements of whole plant were higher than the concentrations in roots. The results showed that the plant contents of nitrogen, potassium and zinc were the highest in March, while the concentrations of phosphorus, calcium, iron and copper were in February. The concentrations of magnesium, manganese and copper was the highest in January and April respectively. The values ​​of nitrogen, phosphorous, potassium, calcium, magnesium, iron, manganese, zinc and copper ranged from 11.1 to 18.4 g kg^−1^, 4.17–2.33 g kg^−1^, 13.73–18.97 g kg^−1^, 24.50–28.90 g kg^−1^, 10.40–12.30 gkg^−1^, 1500–1677 mg kg^−1^, 45.45–49.29 mg kg^−1^, 70.70–177.23 mg kg^−1^, 16.78–73.46 mg kg^−1^, respectively. Furthermore, the results exhibited that the lowest values of the elements appeared in the plant roots in April. As well as, the distribution of the elements followed the normal life curve from January to April. Besides that, the evaluated elements satisfy the needs of the grazing animals' life in which this type of plant grows.

## 1 Introduction

The balance between nutrients and minerals is important for the growth and health of animals, especially wild animals such as goats, sheep and camels. Minerals that are very important for all living organizations play an important role in improving the activity of the rumen and increasing the quality of the use of feed in ruminants (Weiss, 2008). Providing a complete diet, including proteins, fats, vitamins, minerals and water, is important for wild animals that live in the desert, such as camels, goats and sheep. Reducing dependence on imported feed is a goal pursued by the Kingdom of Saudi Arabia (Alzarah, 2020). Trace minerals are important for the metabolic activities of livestock and poultry. These functions aid in the growth and development of cattle and poultry, as well as immunological function and reproductive performance. Minerals are necessary for the support of numerous enzyme systems, in addition to boosting reproductive and production performance characteristics.

Trace minerals are essential in the metabolic activities of livestock and poultry. These responsibilities aid in the development and growth of cattle and poultry, as well as immunological function and reproductive success. Minerals are essential for the support of numerous enzymatic systems, in addition to increasing reproductive and production efficiency metrics.

(https://www.kemin.com/in/en/markets/animal/nutritional-efficiency/mineral-nutrition). Furthermore, minerals combine with proteins, lipids, and other substances to build soft and hard tissues of the body, and they have a specific influence on osmotic pressure, acid-base balance, and nerve and muscle stimulation by connecting the structure of enzyme and hormone systems (Eren, 2009). According to Altıntas (2013), the absence or excess of minerals in animal feed, as well as insufficient or excessive mineral consumption by animals, can have negative impacts on the reproductive, developmental, and immunological systems of animals, in addition to their production. Because the minerals substances play important role in metabolic system of animal, the animal will be taken from grazed plants (Kutlu et al., 2005, Gokkus et al., 2013). Plant mineral contents are one of the mostly dependent on the ecosystem characteristics such as species of pastures, soil types, climate, phonological period and abiotic factors (Underwood and Suttle, 1999). Mineral elements such as Ca, P, K, Fe and Cu are found in high quantitmilleries in pastoral plants that grow in the spring, whereas plants that grow in the autumn contain zinc and manganese in high amounts.

Plants' ability to accumulate mineral substances within their bodies is determined by the plant's development period, nutrient content, root structure, the structure of the soil in the area where the plant grows and its mineral matter content, and the amount and distribution of precipitation during the vegetation period. (Mandal, 1997, Chetri et al., 1999, Abdullah et al., 2013, Temel and Surmen, 2018, Temel, 2019). As a result, it is critical to understand the mineral properties of the plants used as feed sources, as well as their quality features, in order to determine the amount and content of mineral substances that animals would take from the feeds (Keskin et al., 2016). In addition to these characteristics, plant shoots are favoured as an alternative feed source in animal nutrition due to their high nutritional content (Oktay and Temel, 2015a), and their shoots are heavily chewed by animals (Temel and Temel, 2018). Due to its ability to maintain shoot development and greenness during vegetation, it is an important feed source for small ruminants that are grazed, particularly during the summer and autumn seasons (Oktay and Temel, 2015b). To the best of our knowledge, there have been no investigations on determining the macro and micro mineral composition of phog during its active development stage. There has only been one study on determining the mineral content of plants in the spring (February) season (Abdullah et al., 2013). Grazing animals must consume enough forages to meet their mineral requirements. Factors that reduce forage intake (for example, low protein and excessive lignification) also affect overall mineral consumption. Mineral concentrations in plants are affected by factors such as soil type, plant species, maturity stage, dry matter production, grazing management, and environmental climate (Khan et al., 2005, McDowell et al., 1983). In addition, the level of minerals of feedstuff and their biological availability is very important (Dost, 1997, Dost, 2001, Dost et al., 1990). Minerals are required for life to meet the demands of development and production, as well as to replace quantities lost during regular metabolism. Minerals participate in a variety of biological activities as enzyme components and have structural and osmotic roles in a variety of animal tissues (Masters and White, 1996). They added that about 19 mineral elements are essential for animals and other may be essential but the evidence is inconclusive. The essential elements as reported by both authors were calcium (Ca), phosphorus (P), magnesium (Mg), potassium (K), sodium (Na), sulphur (S), cobalt (Co), copper (Cu), iron (Fe), iodine (I), selenium (Se), zinc (Zn), molybdenum (Mo), vanadium (Va), boron (B), lithium (Li), lead (Pb), cadmium (Cd) and Tin (Sn). Dierenfeld et al. (1995) found that the concentration of mineral elements on dry matter basis differed significantly among the 26 plant species they studied. They found that calcium content was 0.55–4.29%, potassium 0.28–1.71%, magnesium 0.12–0.65%, sodium 0.001–0.074%, phosphorus 0.06–0.19%, and zinc 2.5–6.4 Âµg / gram, which were lower or within the dietary level required for feeding dairy animals. However, copper (3.0–12.2 μg / g), iron (2.5–29 μg / g) and manganese (10.9–269 μg / g), which were sufficient for feeding most animals. Miller (1996) showed that zinc, copper, selenium; manganese and potassium contents of plant species within>100 sites in saline lands are much less, than the dietary needs of cows that were estimated in these areas. They also found that the same plants contents of sulfur, magnesium, exceeded the dietary needs of these cows. Al-Zaid et al. (2004) claimed that Panicum turgidum. Forssk. contents of sodium, potassium and magnesium was within the requirements of sheep, goats and camels, while its content of phosphorus and micro elements (zinc, copper, manganese) were low.

For sustainability and reducing dependence on importing fodder from abroad or cultivating varieties that consume water with limited resources. It is necessary to search and find natural resources such as wild plants that exist in the desert environment of the Kingdom of Saudi Arabia that can live in harsh conditions from high temperatures, lack and quality of water (Alzarah, 2020). There is a wide gap between the nutritional needs of animal feed and the quantities produced in Saudi Arabia. Therefore, large quantities of feed are imported to reduce the gap between production and consumption.

So this study aims to follow the monthly changes of minerals in different parts of *Calligonum polygonoides* L. ssp. *comosum* (L’Hér.) plant to determine what is aerial plant parts has optimum levels of the minerals which meet the requirements of grazing ruminan in wild desert.

## 2 Materials and methods

### 2.1 Studied area

The vegetative aerial parts of *Calligonum* comosum wild plant which grown in different sits at Research and Tanning Station of king Faisal University, which is protected since 1975. The study area located in the Eastern Province of Saudi Arabia, between 25°16′14″and 25°15′25″N and 49° 43′20″ and 49°41′42″ E with the area of 6 km^2^ (600 ha). The raise of the area is 150 m above sea level. The studied area is located in tropical zone with a hot desert climate. The average annual rainfall in the region is 72 mm and annual evapotranspiration potential is 3600 mm. The average annual temperature is 21.46 °C with range between 2.20 and 42.37 °C. The soil order is arid soil.

### 2.2 Preparation plant sample for measuring of elements

The plants were collected monthly from Jnuary to April/2020G and divided into the vegetative (leaves and Stems and the whole plant as aerial part) and grouped according to each month and part. The plant samples were cleaned from dust with brush then washed with 0.1 M HCl and following the samples were washed three times by using deionized water. After that, the samples were air dried for 48 h. The samples were dried for two days at 65 °C in dried oven and then grinded and sieved in mesh sieve No. 60, after which the samples were stored in plastic bags until the content of the elements were estimated.

### 2.3 Estimation elements content of plant

0.5 g of dried plant sample has been digested in 50 ml volumetric flask with 2.5 ml of concentrated sulfuric acid (H_2_SO_4,_ 95–97%) on hotplate at approximately 270 °C. Repeated additions of H_2_O_2_ were added until the digest become clear (Cottenie, 1980). After digestion, deionized water was added to the final volume of 50 ml in a volumetric flask. Elements were determined in the liquid sample by using Atomic absorption and emission spectrometer model Shimadzu-AA7000. While the total nitrogen in the samples was determined according to Cottenie (1980) by using micro kjeldahl method and phosphor.

The obtained data were analyzed using SAS computer program. Means were differentiated by using LSD test as described by Snedecor and Cochran (1989) was analysed as per the Khan et al. (2019) studies.

## 3 Results

### 3.1 Analysis of variance of the minerals content

Minerals (macro elements and microelements) content was estimated monthly in the aerial parts of the plant (whole plant, leaves, and stems). Analysis of variance is shown in Table 1, Table 2, Table 3, Table 4, Table 5. Results illustrated that significant monthly changes were found in the recording to ash, N, P, K, Ca, Mg, Mn, Zn, and Cu. Whereas significant variations of aerial parts were found in their contents of ash, N, P, K, Ca, Mg and Cu. However, the interaction between months and different parts was significant only on ash%, C, P, K, and Cu.

**Table 1:** The change of ash%, N, P, K, Ca and Mg g kg^−1^ in plant parts (whole plant, leaves, and stem) of ***Calligonum comosum*** through Months (January, February, March and April of 2020G).

Treatments	*Ash%*	*N*	*P*	K	Ca	Mg
						*g kg^−1^*
***Months (M)***						
January	12.75^b^	14.69^a^	3.38^a^	16.38^b^	27.20^b^	11.62^a^
February	14.31^a^	15.31^a^	3.43^a^	16.32^b^	28.00^a^	11.57^a^
March	7.22^c^	16.74^a^	3.34^a^	17.01^a^	27.16^b^	11.20^a^
April	7.82^c^	14.21^a^	2.97^b^	14.86^c^	26.29^c^	10.62^b^
*LSD_0.05_*	1.33	NS	0.18	0.421	0.484	0.412
Probability	<10^-4^	>0.05	<10^-4^	<10^-4^	<10^-4^	<10^-4^
***Plant parts (PP)***						
Whole plant	11.06^a^	17.3^a^	3.13^b^	16.01^b^	27.82^a^	11.32^b^
leaves	11.98^a^	16.4^a^	4.01^a^	17.55^a^	28.05^a^	11.84^a^
Stem	8.54^b^	12.0^b^	2.70^c^	14.87^c^	25.60^b^	11.16^b^
LSD_0.05_	0.317	2.87	0.16	0.365	0.419	0.356
Probability	<10^-4^	<10^-3^	<10^-4^	<10^-3^	<10^-4^	<10^-3^
***Interaction between M and PP***						
M × PP	****	NS	*	**	NS	NS
LSD_0.05_	2.29	0.57	0.16	0.728	0.076	0.071
Probability	<10^-3^	>0.05	<0.05	<0.01	>0.05	>0.05
CV%	12.90	22.20	5.58	26.7	18.23	3.68

Means in the same column followed by different letters are significantly different at p < 0.05.

*,**,***,**** indicates significance at the 0.05, 0.01,0.001 and 0.0001 levels, and NS means insignificant at level p < 0.05. LSD_0.05_ least significant difference at 0.05 level of significance. Probability < 0.05, 10^-2^, 10^-3^ and 10^-4^ means the probability of signification, CV means coefficient of variation.

**Table 2:** The change of Fe mg kg^−1^ in plant parts (whole plant, leaves, and stem) of ***Calligonum comosum*** through Months (January, February, March and April).

Treatments Months	Fe (mg kg^−1^) in Plant parts			
	Whole plant	leaves	Stem	$Means-M
January	1610	1537	1510	1552a
February	1677	1601	1573	1617a
March	1599	1621	1573	1598a
April	1616	1587	1500	1619a
LSD_0.05_	NS			77.4
$$Mean-plant parts	1625a	1586a	1578a	
LSD_0.05_	67.1			
CV%	4.96			

∂LSD0.05: The least significant differences at p < 0.05. CV is coefficient of variance.

$ The same letters on the columns of months treatment show insignificant differences at p < 0.05.

$$ The same letters on the row of plant parts treatment show insignificant differences at p < 0.05.

**Table 3:** The change of Mn mg kg^−1^ in plant parts (whole plant, leaves, and stem) of ***Calligonum comosum*** through Months (January, February, March and April).

Treatments Months	(Mn, mg kg^−1^) Plant parts			
	Whole plant	leaves	Stem	$Means-M
January	35.19	37.65	38.72	37.18b
February	36.65	39.21	40.33	37.18b
March	43.37	41.08	40.12	41.52b
April	47.95	49.29	45.45	47.78a
LSD_0.05_	NS			5.08
$$Mean-plant parts	40.95a	41.81a	41.16a	
LSD_0.05_	4.40			
CV%	12.57			

∂LSD0.05: The least significant differences at p < 0.05. CV is coefficient of variance.

$ The same letters on the columns of months treatment show insignificant differences at p < 0.05.

$$ The same letters on the row of plant parts treatment show insignificant differences at p < 0.05.

**Table 4:** The change of Zn mg kg^−1^ in plant parts (whole plant, leaves, and stem) of ***Calligonum comosum*** through Months (January, February, March and April).

Treatments Months	Zn (mg kg^−1^) in Plant parts			
	Whole plant	leaves	Stem	$Means-M
January	9.69	13. 06	12. 04	12. 04ab
February	10.92	13. 61	13. 27	12. 54ab
March	13.56	13.43	17.72	14. 90a
April	13.44	12. 30	7. 07	9. 71b
LSD0.05	NS			4.75
$$Mean-plant parts	10.86a	13.01a	13.03a	
LSD0.05	4.11			
^∂^CV%	39.46			

∂LSD0.05: The least significant differences at p < 0.05. CV is coefficient of variance of the species examined is in the defined range and at the level of meeting Zn requirements of the animals recommended by NRC.

$ The same letters on the columns of months treatment show insignificant differences at p < 0.05.

$$ The same letters on the row of plant parts treatment show insignificant differences at p < 0.05.

**Table 5:** The change of Cu mg kg^−1^ in plant parts (whole plant, leaves, and stem) of ***Calligonum comosum*** through Months (January, February, March and April).

Treatments Months	(Cu, mg kg^−1^) Plant parts			
	Whole plant	leaves	Stem	$Means-M
January	45.66	66.35	73.46	61.82a
February	52.39	73.21	67.54	64.38a
March	52.82	34.42	52.88	46.71b
April	21.87	16.78	24.63	21.09c
LSD_0.05_	5.170			9.36
$$Mean-plant parts	43.19b	47.69ab	54.63a	
LSD_0.05_	8.10			
CV%	19.73			

∂LSD0.05: The least significant differences at p < 0.05. CV is coefficient of variance.

$ The same letters on the columns of months treatment show insignificant differences at p < 0.05.

$$ The same letters on the row of plant parts treatment show insignificant differences at p < 0.05.

### 3.2 Nitrogen (N g kg^−1^)

**Fig. 1:**
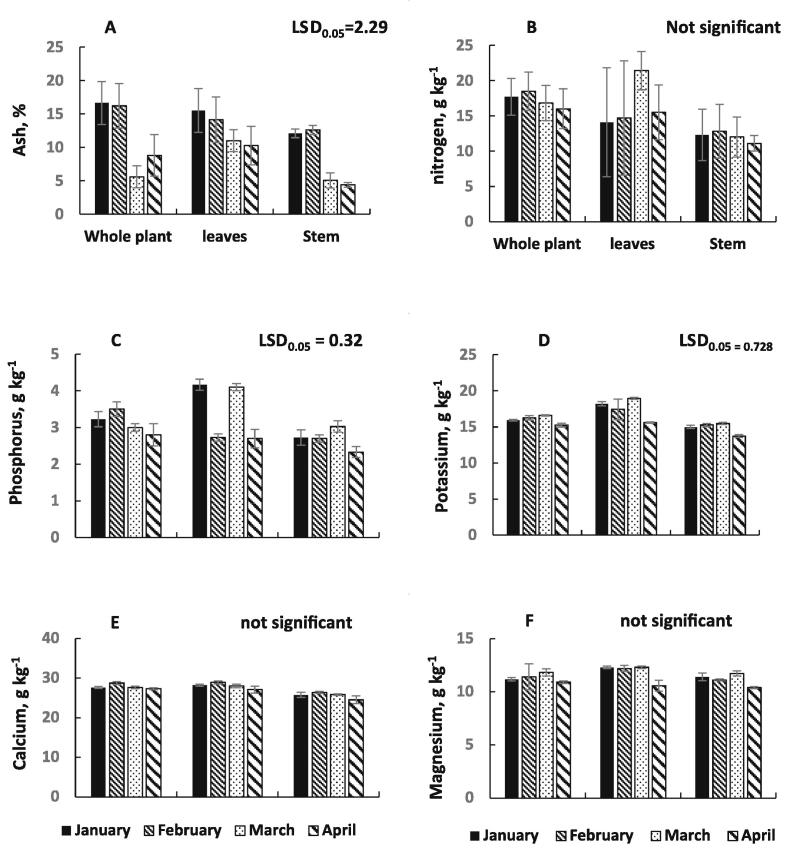
A: Ash (%), B; Nitrogen (g kg^−1^), C: phosphorus (g kg^−1^), D: potassium (g kg^−1^), E: calcium and F: magnesium (g kg^−1^) in plant parts (whole plant, leaves, and stem) of ***Calligonum comosum*** through Months (January, February, March and April of 2020G). LSD_0.05_ is the least significant difference. Not significant is insignificant differences at p < 0.05. Strips of columns mean standard deviation of error.

Nitrogen is the source of protein for many organisms such as plants and plays a good role in determining the quality of feed as food for animals. The analytical results of nitrogen g kg^−1^ are presented in Table 1. As a result of the total N through the studied months, it appeared that the N content did not vary significantly in which the content ranged from 14.21 g kg^−1^ in April and 16.74 g kg^−1^ in March. The results of means comparison of N were obtained highly value in whole plant (17.30 g kg^−1^) and lower content in stem (12.0 g kg^−1^) while the content was 16.4 g kg^−1^ in leaves without no significant variation between the whole plant and leaves. The results in (Fig. 1) presented that the highly nitrogen content was 18.4 g kg^−1^ in whole plant through February and lower value was in stem at April.

### 3.3 Phosphorus (P, g kg^−1^)

The relevance of phosphorus (P) to plants, grazing animals, and humans is undeniable. Phosphorus (P) is essential for plant growth and can be found in all living plant cells. It is involved in various critical plant operations, such as energy transfer, photosynthesis, sugar and starch processing, nutrient flow within the plant, and the transmission of genetic traits from one generation to the next. Table 1 shows the phosphorus content changes in different aerial parts through studied months. By way of a result of the phosphorus the studied months, it appeared that the P content vary significantly in which the content ranged from 3.43 g kg^−1^ in February and 2.97 g kg^−1^ in April without any significant through first three months from study. By looking at the phosphorous changes in the plant parts, the results recorded that the phosphorous content of the whole plant (4.01) > leaves (3.13) > stem (2.70 g kg^−1^) with significant variations in the three aerial parts of the plant. Significant differences were found in phosphors content in plant under the effect of months and aerial parts (Fig. 1). The highest content of phosphorus (4.17 g kg^−1^) was found in leaves at January while the lowest content (2.70 g kg^−1^) was in stem at April.

### 3.4 Potassium (K, g kg^−1^)

Generally, potassium content in this plant ranged between 13.73 in stem of April and 16.60 g kg^−1^ in whole aerial plant at February with significant differences between the values under the effect of months and aerial plant.

### 3.5 Calcium (Ca, g kg^−1^)

The amount of Ca in the *Calligonum comosum* wiled plant in four months from January to April is given in Table 5. It has been determined that there are significant changes in Ca mineral contents of the plant according to months and plant parts while the change through the months in different parts of the plant is not significant (Fig. 1). Calcium content of the plant changed by 26.29 in April to 28.0 g kg^−1^ in February. According the changes through months the Ca values increased from January to February and then decreased.

### 3.6 Magnesium (Mg, g kg^−1^)

The Changes of Mg content in *through 4 months from* January to April in different parts of ***Calligonum comosum***

wild plant are publicized in Table 1. According the results the highest value of Mg was found in January (11.62 g kg^−1^) and lower content was found in April (10.62 g kg^−1^) with significant variation between them while no significant differences were recorded between the mean of January, February and March. The results of Table 1 revealed significant differences between aerial parts of the plant under study (whole plant, leaves and stem) with regard Mg content. The high Mg concentration (11.84 g kg^−1^) was found in leaves but the low values (11.16 g kg^−1^) was noted in stem. Concerning changes of Mg content through months and aerial parts (Fig. 1), the highest value (12.30 g kg^−1^) were obtained in leaves through January and March, while the stem produced the least value (10.40 g kg^−1^) in April.

### 3.7 Iron (Fe, mg kg^−1^)

The Fe amounts in different parts of *Calligonum comosum* through four months from January to April are presented in Table 2. Statistical analysis revealed that the iron content showed insignificant differences as to months, aerial plant part and the average iron content through the months ranged between 1552 mg kg^−1^ in January and 1617 mg kg^−1^ in April. The content of Fe follows that order whole plant (162.5 mg kg^−1^) > leaves (1586 mg kg^−1^) > stem (158.7 mg kg^−1^). The higher content was found in whole plant (167.7 mg kg^−1^) at February while the lowest value was in stem (150.0 mg kg^−1^) through April.

### 3.8 Manganese (Mn, mg kg^−1^)

The manganese content of *Calligonum comosum* did not change significantly according to plant parts and parts through four months while the average contents through months were varied significantly (Table 3). The average manganese contents during the 4-months January, February, February and April were determined as 37.18 mg kg^−1^, 37.1 mg kg^−1^, 41.52 mg kg^−1^ and 47.78 mg kg^−1^, respectively (Table 3) without any significant varied between the content of January, February and March. The Mn concentration in leaves (41.81 mg kg^−1^) was higher than of both stem (41.16 mg kg-1) and whole plant (40.95 mg kg-1). Also, Table 3 revealed that the lower levels of Mn was 35.19 mg kg-1 in whole plant at January while the higher content was 49.29 mg kg-1 in leaves at April without any significant between plant parts and months.

### 3.9 Zinc (Zn, mg kg^−1^)

According the represented results in Table 4 showed that the highest Zn contents (14.90 mg kg^−1^) were obtained from Calligonum comosum in March and lowest values (9.71 mg kg^−1^) was recorded in April, and significance difference was found between two months in terms of Zn content (Table 4). In addition, the studies conducted have revealed that there are no significant variations in Zn contents among the different plant parts of *Calligonum comosum*. The content of Zn fallows this order stem (13.03 mg kg^−1^) > leaves (13.01 mg kg^−1^) > whole plant (10.86 mg kg^−1^).

### 3.10 Cupper (Cu, mg kg^−1^)

Cu content of *Calligonum comosum* changed significantly according to plant parts and through four months (Table 5). The average Cu contents during the 4-months January, February, February and April were determined as 61.82 mg kg^−1^, 64.38 mg kg^−1^, 46.71 mg kg^−1^ and 21.09 mg kg^−1^, respectively (Table 3) without any significant varied between the content of January, and February.

## 4 Discussion

These results are in the context of the findings of Bidgoli (2018) in their study on the forage quality of *Calligonum* comosum in three phonological growth stages (vegetative, flowering and seedling). While Khan et al. (2005) showed that the contents of minerals of leaves in different forage and grasses as related to requirements of ruminants are differed from type to type and through growth period. The ash content is a good indicator for total minerals in feedstuffs. The values of ash% according different parts and months and their interactions are presented in Table 1. The obtained results revealed that the ash% was significantly affected by recorded month and plant parts and their interaction. The ash% varied from 7.22 to 14.31%. These values are in the range which found by Khan et al. (2005) where they indicated that the forage normally contain 3% to 12% ash on a dry matter basis. Concerning month effect the highest value (14.31%) was obtained at February while the lower value (7.22%) was at month of March. These results n agreement with Alzarah (2020) who found that the high ash% value was produced (15.47%) in February, whereas the least ash value% (6.81%) produced in May. Regarding plant parts, the data of Table 1 revealed that the highly content of ash (11.98%) in leaves while the lower value was recorded in stem (8.54%). That means the minerals are moved from roots to stem and accumulated in leaves and aerial whole plant rather than stem. Significant difference was found in the effect of interaction between months and aerial plant parts on ash% (Table 1&Fig. 1). The highest ash% (16.63%) was found in the aerial whole plant in January. The least result (4.40%) was estimated in stem at April.

The N content increased as the months progressed from January to March, and then began to decline in April. This result confirms what Batooli, (2011) referred to his study as he showed the quality of feed varies from the period growth of the plant from March to April. The N content increased as the months progressed from January to March, and then began to decline in April. This result confirms what Batooli, (2011) referred to his study as he showed the quality of feed varies from the period growth of the plant from March to April.

The phosphorous results confirmed the gotten of Dongmei et al. (2005). The phosphorus content of *Calligonum comosum* is above the ranges of different natural growing plant species that the varied between 1.3 and 4.4 g kg^−1^ (NRC, 2001). Alzarah (2020) in his study on determination on mineral elements of halophytic plant species in Eastern province of Kingdom of Saudi Arabia indicated that the Calligonum plant has 3.4 ± 0.5 g kg^−1^. That means the leaves has higher content in January. This percentage is higher in most plant parts of Calligonum comosum except in stem in March than alfalfa (2.8 g kg^−1^) according to NRC (2016a).

The absence of K leads to the weakness in the bone of animals and a decline in their growth and development as indicated by NRC, 2001, McDonald et al., 2011, Kutlu et al., 2005. Fig. 1 shows the changes of potassium content in different months and also in different aerial parts of *Calligonum comosum* wild plant.

It was higher than the content of studied plant. But it was higher than the minimum requirement for cattle (6 g kg ^-1^), sheep (5–8 g kg^−1^) and milking cows (10–10.5 g kg^−1^) but lower than the highest level that could be used for dry cattle (30 g kg^−1^) (NRC, 2001, NRC, 2016b). The results of Table 1 revealed significant differences between the mean of K content through months (from January to April) where the low content (14.86 g kg^−1^) was in April and higher value reached to 17.01 g kg^−1^ in March. This result is in agreement with that obtained by Temel (2019) also by Al-Jaloud and Hussain (2006) in their study on Sabkha ecosystem and halophyte plant communities in Saudi Arabia they indicated that the K content was 4–28.8 g kg^−1^ in eastern province and 0.5–2.6 g kg ^-1^ while in Western ranged between 0.5 and 2.6 g kg ^-1^. In addition, Alzarah (2020) indicated that the K content of *Calligonum comosum* wild plant from Eastern province was 16.0 ± 3.9 g kg^−1^. The levels of K in different parts of this plant as shown in Table 4 were follow that order: leaves (17.55 g kg^−1^) > whole plant (16.01 g kg^−1^) > stem (14.87 g kg^−1^). The above results summarized that the higher values of K were in leaves grown in March. As whole the level of K in *Calligonum comosum* was lower than alfalfa (35 g kg^−1^). The comparison of K content in *Calligonum comosum* with need requirement the results indicated that it was higher than the minimum requirement the for cattle (6%) (NRC, 2016a) and sheep (5–8%) (NRC, 2016b) and milking cows (10–10.5%) but lower than the highest level that could be used for dry cattle (30%) (NRC, 2016a).

These results confirm in Calcium by Mandal (1997) , Chetri et al., (1999); Abdullah et al., 2013, Temel and Surmen, 2018, Temel, 2019 reached, as they found that the content of the elements varies from month to month, vegetation period and according to the seasons of the year. Our results are consistent with Temel (2019) indicated that the mean calcium content of Calligonum comosum in through seven months from April to October of his study varied from 13.2 to 18.2 g kg^−1^. Concern to calcium changes in the aerial parts of the plant specified that leaves (28.05 g kg^−1^) > All plant (27.82 g kg^−1^) > stem (25.06 g kg^−1^. The higher values were 28.09 g kg^−1^ at February in leaves and the lower value was in stem of April (Fig. 1). These results are in agreement with that obtained by Alzarah (2020) in their study on the Ca content of Calligonum comosum where they found that the Ca contents were 26.8 ± 19.2 g kg^−1^. Whereas he reported that these levels were higher than that required by beef cattle (0.18–0.44%), milk cows (0.60–0.65%), sheep (0.25–0.84%) and goats (0.138%) (NRC, 2016b and 2001). However, according to Wardeh (1997), there was no problem for calcium deficiency in camels grazing in the natural pasture. While, Weiss (2008) indicated that the maximum tolerable levels (MTL) of Ca is 1.5% of dietary DM (approximately 2 times the NRC requirement for dairy cows). Al Noaim et al. (1991) in their study of chemical composition of range plants in the Eastern Province of Saudi Arabia showed that the concentration of Ca varied from 8.4 to 23.6 g kg^−1^. The results of Table 1 and Fig. 1 summarized that the Ca of ***Calligonum comosum*** approached from the needing of wild animals according to NCR (2016b) and the higher values were found in leaves through four months of the study.

Magnesium is present in the animal body at a concentration of 0. 4 g kg^−1^ (Kutlu et al., 2005). Magnesium is closely related to calcium and phosphorus. The skeletal structure contains 70% of the magnesium in the animal body, with the remaining amount found in soft tissues and liquids. Magnesium and phosphate are essential for transferase enzyme activity, glucose and lipid metabolism, and cell respiration. A deficiency of magnesium in the animal diet can cause irritability, myotonic and nerve overextension, deficits in glucose and lipid metabolism, weakness in skeletal and hoof development, and a decline in animal growth and development. (NRC, 2001, McDonald et al., 2011, Kutlu et al., 2005). According to NCR (2016b) the level of Mg in Calligonum comosum was higher than in alfalfa (3–10 g kg^−1^) and also higher than that needed for milking cows (1.8–2.1%) (NRC, 2001, NRC, 2016a), sheep (1.2–1.8%) (NRC 2016a), goats (4% −8%) (NRC, 1985, 2016a) and horses (8 g kg^−1^)(NCR2001). Alzarah (2020) determined the Mg content in Calligonum comosum with 11.8 ± 1.5 g kg^-1^and this value was in range of our study (Table 1). From these results, we conclude that eating any part of the plant during the study months meets the nutritional needs of wild animals.

The Fe results are in line with result Alzarah (2020) which indicated that Fe content of *Calligonum comosum* from Eastern province of Saudi Arabia was 211.66 ± 45.39 mg kg^−1^ while Temel (2019) discovered that the Calligonum comosum plant's shoots contained 99.73 mg kg^−1^ to 190.43 mg kg^−1^ throughout a 7-month period. The maximum iron level (190.43 ppm) was found in April, while the lowest iron content (99.73 ppm) was found in October, with iron content decreasing as the development period advanced (Table 3). The Fe content of *Calligonum comosum* was higher than that of alfalfa (189 mg kg^−1^) (NCR, 1985). They are also higher than the levels required for sheep feeding (30–50 ppm) (NRC, 1985), dry cows (50 ppm) (NRC, 2016b), milking cows (1.8–12.8 ppm) (NRC, 2001) and the proposed level for feeding large and small camels (50–100 ppm, respectively) (Wardeh, 1997). The iron level is also lower than the level of 1000 ppm that is needed by cows (NRC, 2001) and camels (Wardeh, 1997) and the level of 500 ppm needed for sheep (NRC, 1985).

The levels of Mn concentration were lower the value which recorded by Alzarah (2020) in *Calligonum comosum* which grown in Eastern province in Saudi Arabia was 65.28 ± 35.88 mg kg^−1^. The difference between Alzarah values and our result may due to location, soil and timing of sampling. These recorded levels in natural plant were generally higher than those of alfalfa (30.3 ppm) (NRC 2016b). They were also higher than the minimum requirements for sheep (20–40 ppm) and dry cows (20 ppm) (NRC, 2016b) and milking cows (14 ppm) (NRC, 2001). Wardeh (1997) suggested a mean level of 40 ppm as a minimum requirement for camels, but most animals could tolerate very high concentrations of manganese up to 1000 mg kg^−1^ for cattle, sheep and camels (Wardeh, 1997). NRC (1985) reported that toxic level of this element is 1000 mg kg^−1^. According to these results, Mn content of *Calligonum comosum* was found to be lower than these critical values, and it has been demonstrated to be sufficient for ruminants. The deficiency of Mn leads to abnormal development and bone disorder in animals (Ayan et al., 2006).

Zinc element takes over an important duty in the activation of enzymes. In addition, the deficiency of Zn may lead to anemia, negative effects on immune system and infertility (Hidiroglou and Knipfel, 1984). Alzarah (2020) indicated the value of Zn was 7.29 ± 2.19 mg kg^−1^ in Calligonum comosum. The recorded data in Table 9** revealed that the Zn concentration of studied plant was less than that of alfalfa (18.6 mg kg^−1^)(NCR, 2016b) and lower than the minimum requirement for sheep (20–30 mg kg^−1^) and milking cows (43–55 ppm) (NRC, 2016b) and camels (40 ppm) (Wardeh, 1997). However, some plant species could provide the minimum dietary requirement for cows (30 ppm) (NRC, 2016b). This content was also far from the maximum permissible limit that could cause toxicity to the animal, which is 500 and 700 ppm for cattle and sheep respectively (NRC, 2016a). Therefore, this study showed that the Zn content of the species examined is in the defined range and at the level of meeting Zn requirements of the animals recommended by NRC.

The Cu concentration in stem (54.63 mg kg^−1^) was higher than of both leaves (47.69 mg kg^−1^) and whole plant (43.19 mg kg^−1^). Also Table 5 revealed that the lower levels of Cu was 16.78 mg kg^−1^ in leaves at April while the higher content was 73.46 mg kg^−1^ in stem at January with significant between plant parts through the months. The levels of Cu concentration was lower than the value which recorded by Alzarah (2020) in ***Calligonum comosum*** which grown in Eastern province in Saudi Arabia was 54.97 ± 4.18 mg kg^−1^. The difference between Alzarah values and our result may due to location, soil and timing of sampling. These recorded levels in ***Calligonum comosum*** plant were generally higher than those of alfalfa (5.3 mg kg^−1^) (NRC 2016b) and are significantly higher than the minimum requirements for sheep (5–11 ppm) (NRC, 1985), cattle (10 ppm) (NRC, 2016a) and milking cows (11 ppm) (NRC, 2016b). However, Wardeh (1997) stated that it is difficult to determine the camels minimum copper requirements because the absorption copper rate depends mainly on its interaction with molybdenum, sulphur and possibly some other elements. In addition, Underwood and Suttle (1999) noted that copper absorption is influenced by climatic factors as well. However, these levels were very high than the toxicity level for sheep (25 ppm) (NRC, 1985) but were lower than the reported toxicity for dry cows and milking cows (100 and 80 ppm) (NRC, 2016a and 2001), respectively.

## 5 Conclusion

The macro and micro- minerals were varied significantly according to aerial parts of Calligonum comosum plant and as to depend months. The nitrogen, phosphorus and potassium content changed significantly according to aerial parts of Calligonum comosum wild plant where a whole plant > leaves > roots, while the concentration of calcium, magnesium, manganese, zinc and copper elements in the leaves was significantly higher than other parts (whole plant > roots). The plant content of nitrogen, potassium and zinc found to be high in March. The measured concentration of phosphorus, calcium, iron and copper are relatively higher during February when the temperature begin to be high, while the concentration of magnesium, manganese and copper was the highest in January and April respectively. It is observed that the plant can meet with the daily N, P, K, Ca, Mg, Fe, Zn, Cu and Mn requirements of small ruminant throughout its 4-month growth period of this work.

## Declaration of Competing Interest

The authors declare that they have no known competing financial interests or personal relationships that could have appeared to influence the work reported in this paper.
